# Comprehensive Analysis of the Expression and Prognosis for Laminin Genes in Ovarian Cancer

**DOI:** 10.3389/pore.2021.1609855

**Published:** 2021-08-25

**Authors:** Bowen Diao, Ping Yang

**Affiliations:** Department of Gynecology, First Affiliated Hospital, School of Medicine, Shihezi University, Shihezi, China

**Keywords:** ovarian cancer, metastasis, laminins, cancer prognosis, bioinformatical analysis

## Abstract

Survival is low in ovarian cancer (OC). Most OC patients demonstrate advanced metastases, and recurrence is common. Dysregulation of laminin interactions is associated with cancer development. However, it is unknown whether laminin subunits can be considered as biomarkers for OC diagnosis, prognosis, and treatment. We used cBioPortal, GEO, ONCOMINE, GEPIA, Human Protein Atlas, Kaplan-Meier Plotter, TIMER, and Metascape to determine the associations among laminin expression, prognosis, and immune cell infiltration in OC. LAMA5, LAMB3, and LAMC2 mRNAs and LAMA3, LAMB1/B2/B3, and LAMC1/C2 proteins were overexpressed in OC tissues compared with normal ovaries. LAMA4, LAMB1, and LAMC1 mRNA upregulation was positively correlated with worse overall survival (OS) and progression-free survival (PFS) in OC. Elevated LAMA2 and LAMC2 mRNA expression levels were related to better PFS or OS, respectively. The results speculated that LAMA5 could potentially be a good prognostic factor in OC. Its expression proves valuable for predicting OS in patients diagnosed with stage Ⅳ and grade 3 OC and PFS in patients diagnosed with all OC stages or grades. LAMB3 and LAMC2 expression was correlated with platinum resistance development. ROC analysis of laminins in OC sets revealed that LAMA2/A4/A5, LAMB1/B2/B3, and LAMC2 could be used to differentiate between malignant tumors and non-neoplastic tissues. LAMA1/A5 and LAMC1 were significantly and negatively correlated with various tumor immune infiltrates (TILs), especially with dendritic cells, CD8^+^ T cells or neutrophil. LAMA4 and LAMB1 might be associated with tumor purity in OC. Overall, LAMA5 and LAMC1 could help predict OC survival and diagnosis and might be deemed important OC oncogenes.

## Introduction

Ovarian cancer (OC) is the eighth most frequently occurring cancer in women worldwide. There were 384,000 OC-related deaths reported in 2018[1]. The median age of patients diagnosed with OC is approximately 60 years, and most patients present with advanced metastasis[2]. The 5-year survival rates for stages I–IV are 93, 68, 27, and 13.4%, respectively [3]. Recurrence is incurable in nearly 75% of all women presenting with advanced disease[4]. Surgical resection and platinum-based chemotherapy are the primary OC treatment strategies. While most patients exhibit favorable and appreciable responses to first-line chemotherapy, cases relapse within 18 months and patients succumb to the disease[2]. Therefore, it is necessary to develop and establish effective markers for the conduction of early diagnosis, for the formulation of strategies addressing metastasis and chemotherapy resistance, and for the discovery of alternatives such as immunotherapy for ovarian cancer.

Previous studies demonstrated the prognostic value of laminins in gastric cancer[5], hepatocellular carcinoma[6], renal cell carcinoma[7], breast cancer[8], colorectal cancer[9], pancreatic cancer, and lung cancer[10]. Laminin is a complex glycoprotein composed of three different polypeptide chains connected by disulfide bonds and is a cross-shaped molecule. Laminins are the chief components of vascular and parenchymal basement membranes and provide structural basis for the extracellular matrix (ECM)[11]. Dysregulated cell laminin interactions are major features of various cancers[11]. Certain members of the laminin gene families are correlated with cancer cell migration and tumor invasiveness. Overexpression of certain laminin chains is associated with cancer progression and poor cancer prognosis[12]. Integrins and nonintegrin molecules are the major cell surface receptors for laminins[13]. At least one laminin gene is detected in approximately half of all ovarian cortical cells. These genes may encode LAMA2/A5, the *β*-chains of LAMB1/B2, the *γ*-chain of LAMC1, and others[14]. However, it is unclear whether the key subunits of laminin genes can serve as biomarkers for OC diagnosis, prognosis, and treatment. Thus, we designed and executed the present study to address gaps in the existing research and to ascertain the potential applicability of laminin genes in various aspects of ovarian cancer treatment.

## Materials and Methods

### cBioPortal

CBioPortal (http://www.cbioportal.org/) is widely used to investigate and visualize multidimensional cancer genomics data[15]. The types of comprehensive gene data in CBioPortal include those pertaining to somatic mutations, DNA copy number changes, mRNA and microRNA expression, among others. A dataset comprising information derived from 307 patients with ovarian serous cystadenocarcinoma contained mRNA data (RNA Seq V2) (TCGA, Firehose Legacy) and was selected to analyze genetic variation. In the present study, mutations, putative copy number alterations from GISTIC, and mRNA expression z-scores (RNA Seq V2 RSEM) were selected for genomic profiles. The data on the relationships between CNA (relative linear copy number) and the mRNA expression of each laminin member were plotted. A Kaplan-Meier plot provided insights into laminin gene mutations and their relationships with OS and DFS in OC patients. A log-rank test was used to determine the significance of the divergence in the survival curve. When *p* < 0.05, divergence was considered significant.

### Oncomine Database

Oncomine (http://www.oncomine.org) is an microarray database of carcinoma and comprehensive platform intended for mining information about cancer genes [16]. Oncomine contains cancer mutation profiles, gene expression data, and relevant clinical information that can be used to identify new biomarkers or therapeutic targets. It combines RNA and DNA-seq data from GEO, TCGA, and reported literature sources. In our study, we obtained the transcriptional expression of laminins between cancer and relevant ovarian tissues. The differences in transcriptional expression were contrasted with the Student’s t-test, and the following parameters were set: *p*-value: 0.01, fold change: 1.5, gene rank: all, data type: mRNA, analysis type: Cancer vs. Normal analysis.

### The GEPIA2 (Gene Expression Profiling Analysis)

The GEPIA2 (http://gepia2.cancer-pku.cn/) web server is a reference source used for gene expression analyses. It is based on the Cancer Genome Atlas (TCGA) and the Genotype-Tissue Expression (GTEx) databases [17]. In the present study, the threshold of |log2FC| was 1, the *p*-value cutoff was 0.05. Spearman’s correlation coefficients were calculated. The function Similar Genes Detection was used to search for genes similar to those encoding laminins.

### The Human Protein Atlas

The Human Protein Atlas (https://www.proteinatlas.org) contains data on nearly 20 types of cancer based on immunohistochemical (IHC) expression[18]. It furnishes tissue and cell distribution information on 24,000 human proteins and is available in the public domain. In the present study, laminin expression in OC and normal tissues was compared via IHC. Antibody names and stain intensities are indicated in the figures.

### The Kaplan-Meier Plotter (Ovarian Cancer)

The Kaplan-Meier plotter database (http://kmplot.com/analysis/) is an analytical prognostic database used for the analysis of malignant tumors. It is used to assess the impact of over 54,000 genes in the prognosis of 21 different types of cancer[19]. In medical training and scientific research, this database is used to explore the relationships between gene expression and tumor prognosis. The Kaplan-Meier plotter bioinformatics analysis platform can be used to inquire into the prognostic value of 12 laminin genes. Based on their median mRNA expression levels, tumor patients were separated into two groups using the Kaplan-Meier plotter and were verified based on K-M survival curves. The risk ratio (HR) and the log-rank *p*-values of the 95% confidence interval (CI) were used to determine OC patient OS and PFS. *p* < 0.05 was considered statistically significant.

### TIMER (Tumor Immune Estimation Resource)

The algorithm in TIMER (https://cistrome.shinyapps.io/timer/) is used to systematically analyze immune cell infiltration in cancer [20]. The associations between each laminin member and massive immune infiltration in OC were investigated. The immunocytes involved included B cells, CD4^+^ T cells, CD8+ T cells, neutrophils, macrophages, and dendritic cells. Spearman’s correlation coefficients were calculated.

### GEO Database and GEO2R

GEO is a communal functional genomic database, which includes a large number of array and sequence data [21]. The two gene chip profiles GSE131978[22] and GSE58470 datasets [23] were obtained from NCBI-GEO. Users can use GEO2R to compare sets of samples in order to classify disparate genes expressed. In this study, the GEO2R online tools were used to distinguish DEGs (Differential expressed genes) between ovarian tumors and adjacent normal tissues, by the cut-off criteria of adjusted *p* < 0.05 and |log2FC| > 1.

### Metascape Analyses

Metascape is a web-based portal that provides inclusive gene list commentary and analysis resources [24]. We selected 20 genes similar to each laminin-family gene from GEPIA2. To identify the biological functions and pathways of these genes, we used Metascape to analyze Kyoto Encyclopedia of Genes and Genomes (KEGG) pathway enrichment. Gene Ontology (GO) term enrichment analysis was performed to determine the functions of the target host genes in terms of biological process, cell composition, and molecular function.

### GeneMANIA and STRING Analyses

We used the GeneMANIA (http://www.genemania.org/) and STRING (https://string-db.org) [25] databases to explore the gene–gene and protein–protein interaction networks of the laminins. GeneMANIA can use large functionally linked data to identify other genes related to target genes. STRING is used to search for known protein interactions. Interactions revealed by STRING are primarily based on the confidence score (reliability index) as well as other collateral information such as protein domains and three-dimensional protein structures.

## Results

### Genetic Mutations in Laminins and Their Associations With Overall Survival and Disease-free Survival in Patients With Ovarian Cancer

**FIGURE 1:**
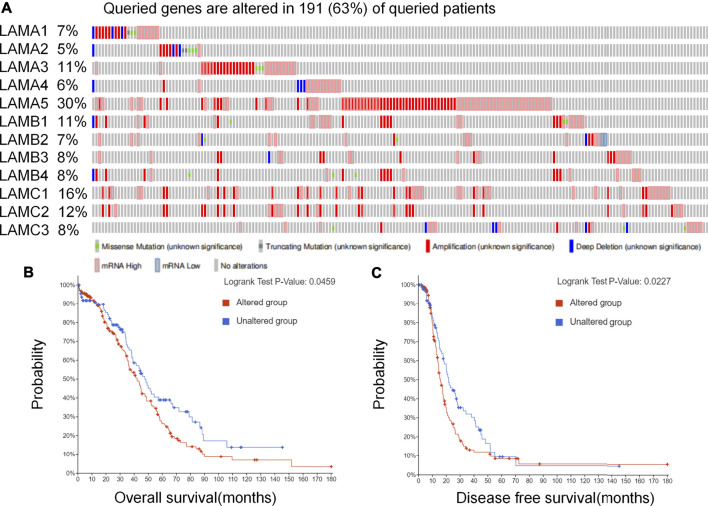
Laminin gene expression and mutation analysis in OC (cBioPortal). **(A)** Laminin gene expression and mutation analysis in OC (cBioPortal) **(B–C)** Genetic alterations in laminins associated with shorter OS and DFS in OC patients.

We evaluated genetic alterations in laminins and their relationships with the OS and DFS of OC patients registered in the cBioPortal database. Figure 1A depicts high laminin mutation rates in the OC samples. Laminin gene expression levels were altered in 191 samples obtained from 303 ovarian cancer patients (63%), and LAMA5 was observed to be the most frequently mutated gene (30%). Other genes with high mutation rates were LAMC1(16%), LAMC2 (12%), LAMA3 (11%) and LAMB1 (11%). Laminin gene amplification and mRNA alteration occurred relatively more often in OC patients. The LAMB2 and LAMC1 mRNA expression levels were significantly and positively correlated with their relative linear copy number alteration (CNA) values. However, the LAMA1/A3/A5, LAMB3, and LAMC2 mRNA expression levels were only weakly correlated with their relative linear CNA values (Supplementary Figure S1). The foregoing results indicated that genetic changes in laminins were correlated with poor OS (Figure 1B, *p* = 0.0459) and DFS (Figure 1C, *p* = 0.0227) in OC patients. They also demonstrated that changes in laminin genes and copy numbers occurred in certain patients with OC and might be related to poor prognosis.

### Aberrant Laminin mRNA Expression in Ovarian Cancer

**FIGURE 2:**
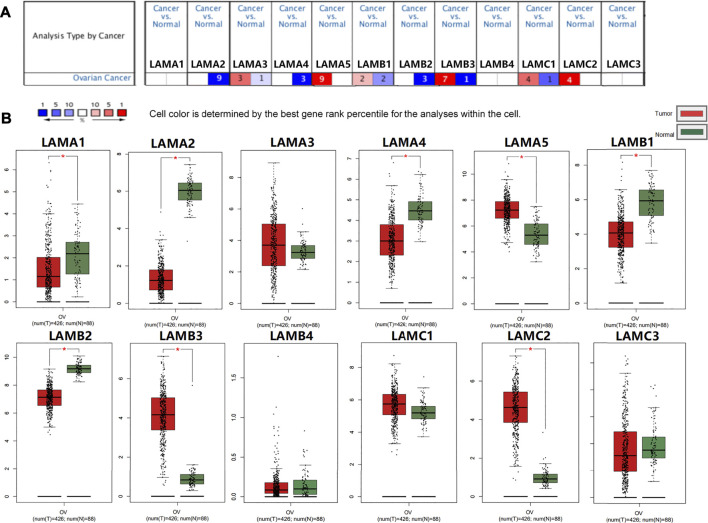
Expression of laminin members in normal ovary and OC tissues. **(A)** Transcriptional expression of Laminins in OC (ONCOMINE database). **(B)** Laminin expression in OC (GEPIA2). *p < 0.05; **p < 0.01; ***p < 0.001; ****p < 0.0001. The red columns represented tumor tissues, and the green column represented normal tissues.

The mRNA expression levels of 12 laminin members were evaluated for OC patients registered in the ONCOMINE database. ONCOMINE includes data on extensive independent studies conducted using various patient cohorts. Figure 2A shows that the mRNA expression levels of 12 laminin family members were detected using the ONCOMINE database and were compared against normal tissues. At least one dataset indicated that the expression levels of LAMA5 and LAMC2 mRNAs were significantly upregulated. In contrast, the expression levels of LAMA2/A4 and LAMB2 mRNAs were significantly downregulated. However, certain studies demonstrated that LAMA3, LAMB1/B3 and LAMC1 were highly expressed in OC whereas other reports presented the opposite findings (Supplementary Table S1).

We used GEPIA2 to evaluate laminin mRNA expression in OC and normal ovary tissues. The mRNA expression levels of LAMA5, LAMB3, and LAMC2 were higher, while those of LAMA1/A2/A4 and LAMB1/B2 were lower in OC tissues than normal ovary tissues (Figure 2B). Our findings suggested that LAMA5, LAMB3, and LAMC2 were overexpressed, whereas the expression levels of LAMA2/A4 and LAMB1/B2 were downregulated in OC tissues.

### Expression of 12 Laminin Proteins in Ovarian Cancer Patients

**FIGURE 3:**
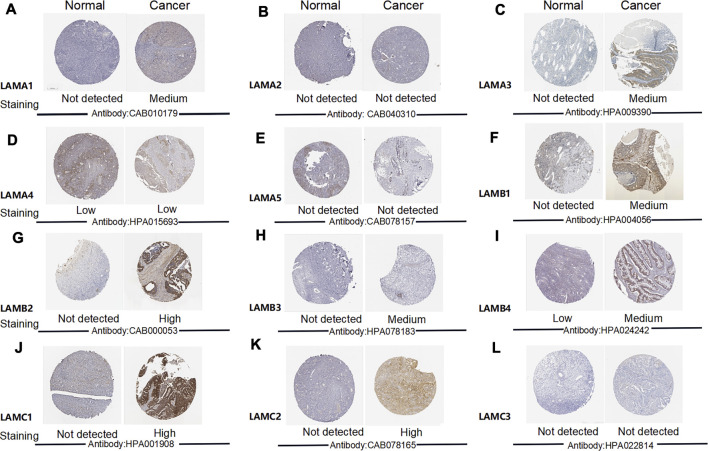
Images depicting IHC analysis performed for distinct laminin family members in OC and normal ovary tissues (the Human Protein Atlas).

We used the Human Protein Atlas (HPA) to explore laminin protein expression patterns in OC (Figure 3). Images depicting the results of immunohistochemical (IHC) staining performed for 11 or 12 samples were used for comparison. This revealed the expression of LAMA1 (six positive, six negative), LAMA2 (zero positive, twelve negative), LAMA3 (eleven positive, one negative), LAMA4 (five positive, seven negative), LAMA5 (one positive, eleven negative), LAMB1 (seven positive, five negative), LAMB2 (seven positive, five negative), LAMB3 (twelve positive, zero negative), LAMB4 (nine positive, two negative), LAMC1 (nine positive, two negative), LAMC2 (twelve positive, zero negative), and LAMC3 (zero positive, 12 negative) laminin proteins. The number of LAMA1-positive and LAMA1-negative cases was similar for the OC samples, but LAMA1 was not expressed in normal ovary tissues. LAMA3 and LAMB1/B3 proteins were expressed at medium or low levels in OC tissues but protein expression was not detected in normal ovary tissues. LAMB2 and LAMC1/C2 were expressed at high, moderate, or low levels in OC tissues but protein expression was not detected in normal ovary tissues. LAMB4 protein was expressed at medium or low levels in OC tissues, but their expression was not detected in normal ovary tissues. The LAMA4 protein expression levels were equally low in both OC and normal ovary tissues. LAMC2 proteins were expressed at high, moderate, or low levels in OC tissues but were not expressed in normal ovary tissues. However, no LAMA2 or LAMC3 protein expression was detected in OC or normal ovary tissues.

We also investigated the associations between laminin mRNA and protein expression. LAMA3 mRNA and protein expression were strongly correlated. There were moderate correlations between the mRNA and protein levels of LAMA1/A2/A4, LAMB1/B3, and LAMC2, LAMA5 and LAMC1 mRNA and protein expression levels were weakly correlated. There was no correlation between LAMB2 mRNA and LAMB2 protein expression, and no data were available for LAMB4 or LAMC3 mRNA and protein correlations (Supplementary Figure S2).

The preceding results suggested that the proteins expression levels of LAMA3, LAMB1/B2/B3, and LAMC1/C2 were higher in OC than normal tissues and there were varying degrees of correlation between the protein and mRNA expression levels of LAMA1/A2/A3/A4/A5, LAMB1/B3, and LAMC1/C2.

### Prognostic Value of Laminin Genes in Ovarian Cancer

**FIGURE 4:**
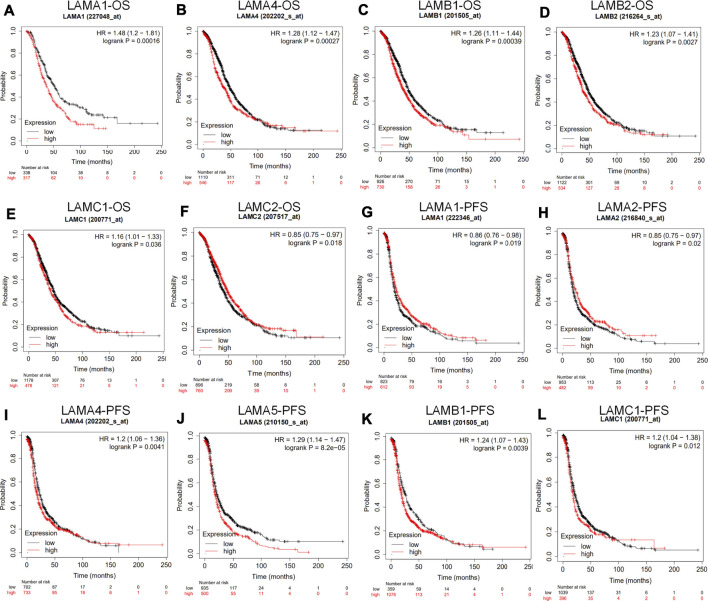
Prognostic value of laminin mRNA levels in ovarian cancer patients (the Kaplan-Meier plotter). **(A–F)** Laminin genes significantly correlated with OS in OC **(G–L)** Laminin genes significantly correlated with PFS in OC.

We used the Kaplan Meier plotter to investigate the impact of laminin mRNA level on survival in patients with OC. Upregulated expression levels of LAMA1/A4, LAMB1/B2, and LAMC1 and downregulated expression levels of LAMC2 were significantly and positively associated with poor OS in OC patients (Figures 4A–F). While upregulated expression levels of LAMA4/A5, LAMB1, and LAMC1 mRNAs were significantly and positively associated with poor PFS, elevated LAMA1/A2 expression levels were significantly and positively associated with better PFS (Figures 4G–L).

In general, the foregoing results showed that increases in LAMA4, LAMB1 and LAMC1 mRNA expression levels were positively related to poor OS and PFS, whereas upregulated LAMA5 mRNA level was positively related to worse PFS. Thus, increased LAMC2 mRNA expression may be considered to predict longer OS and upregulated LAMA1/A2 mRNA level is associated with better PFS in patients with OC.

### Correlation of Laminin Gene Expression With Clinicopathological Features and Prognosis in Ovarian Cancer Patients

We used the Kaplan Meier plotter to investigate the associations among laminin mRNA expression, OS, and PFS in patients at different clinical stages and pathological grades of OC. Table 1 shows that for patients with stage I or stage II OC, the LAMA1 and LAMB1 mRNA expression levels were strongly associated with worse OS (HR > 1), and the LAMA3 and LAMB4 mRNA expression levels were significantly related to better OS (HR < 1). For stage III OC patients, the LAMA1/A4, LAMB1, and LAMB3 mRNA expression levels were markedly associated with unfavorable OS, while the LAMB4 and LAMC2 mRNA expression levels were strongly related to favorable OS. For stage Ⅳ OC patients, transcriptional LAMB3 expression was significantly associated with longer OS, while transcriptional LAMA1/A3/A5 expression were significantly correlated with shorter OS. The data showed that for patients with grade 1 or 2 OC, the LAMA3, LAMB4 and LAMC2 mRNA expression levels were significantly correlated with longer OS; however, the LAMA1/A4 and LAMB1 mRNA expression levels were significantly related to shorter OS. For grade 3 OC patients, the LAMA1/A4/A5 and LAMB1 mRNA expression levels were significantly associated with poor OS.

**TABLE 1:** The relationship between Laminins and OS in different tumor grades and stages of OC (Kaplan–Meier plotter). Bold font indicates significant difference.

Genes	Stage I + II (179 cases)		Stage III (1,079 cases)		Stage IV (189 cases)		Grade 1 + 2 (381 cases)		Grade 3 (1,024 cases)	
	HR (95%CI)	*p*-value	HR (95%CI)	*p*-value	HR (95%CI)	*p*-value	HR (95%CI)	*p*-value	HR (95%CI)	*p*-value
LAMA1	4.66	**0.01**	1.47	**0.0022**	2.15	**0.012**	1.99	**0.0034**	1.57	**0.00039**
	(1.29–16.87)		(1.15–1.88)		(1.16–3.98)		(1.25–3.19)		(1.22–2.03)	
LAMA2	0.3	0.041	1.19	0.079	1.25	0.23	0.75	0.11	1.13	0.16
	(0.09–1.02)		(0.98–1.43)		(0.87–1.81)		(0.52–1.07)		(0.95–1.35)	
LAMA3	0.33	**0.0034**	0.89	0.17	1.53	**0.023**	0.69	**0.018**	1.13	0.15
	(0.15–0.72)		(0.75–1.05)		(1.06–2.23)		(0.5–0.94)		(0.96–1.33)	
LAMA4	1.57	0.28	1.28	**0.0051**	0.84	0.35	1.44	**0.012**	1.23	**0.021**
	(0.68–3.62)		(1.08–1.51)		(0.58–1.21)		(1.08–1.92)		(1.03–1.47)	
LAMA5	1.96	0.088	0.85	0.06	1.72	**0.004**	1.2	0.22	1.25	**0.0082**
	(0.89–4.29)		(0.72–1.01)		(1.18–2.51)		(0.9–1.61)		(1.06–1.49)	
LAMB1	2.57	**0.013**	1.43	**8.7E−05**	0.75	0.15	1.47	**0.0083**	1.32	**0.0034**
	(1.19–5.56)		(1.2–1.72)		(0.51–1.11)		(1.1–1.96)		(1.1–1.59)	
LAMB2	1.77	0.22	1.14	0.13	1.16	0.42	1.28	0.13	0.88	0.16
	(0.71–4.41)		(0.96–1.35)		(0.8–1.69)		(0.93–1.76)		(0.73–1.05)	
LAMB3	0.46	0.075	1.2	**0.04**	0.64	**0.015**	0.81	0.18	1.14	0.15
	(0.19–1.1)		(1.01–1.44)		(0.44–0.92)		(0.59–1.11)		(0.95–1.37)	
LAMB4	0.34	**0.0045**	0.81	**0.027**	0.7	0.08	0.69	**0.024**	0.9	0.23
	(0.16–0.74)		(0.68–0.98)		(0.47–1.05)		(0.5–0.95)		(0.75–1.07)	
LAMC1	1.77	0.22	1.14	0.13	1.16	0.42	1.28	0.13	0.88	0.16
	(0.71–4.41)		(0.96–1.35)		(0.8–1.69)		(0.93–1.76)		(0.73–1.05)	
LAMC2	0.35	0.078	0.83	**0.041**	1.37	0.1	0.7	**0.02**	0.89	0.19
	(0.11–1.18)		(0.69–0.99)		(0.94–2.01)		(0.51–0.95)		(0.75–1.06)	
LAMC3	1.48	0.34	1.11	0.28	1.43	0.055	0.8	0.12	1.15	0.11
	(0.65–3.34)		(0.92–1.33)		(0.99–2.05)		(0.59–1.06)		(0.97–1.37)	

Table 2 shows that the LAMA2/A3/A4, LAMB1 and LAMC2 mRNA expression levels were significantly associated with longer PFS in stage I or stage II OC patients, while the LAMA5 mRNA expression levels was significantly related to shorter PFS in the same patients. In stage III OC patients, the LAMA4/A5, LAMB1/B2, and LAMC1/C3 mRNA expression levels were significantly related to poor PFS. The LAMA3 and LAMA5 mRNA expression level were significantly correlated with unfavorable PFS in stage IV OC patients. The LAMA2/A3 and LAMC2 mRNA expression levels were markedly correlated with longer PFS in grades 1 and 2 OC patients, whereas the LAMA4 and LAMA5 mRNA expression levels were significantly associated with shorter PFS in grades 1, 2, and 3 OC patients. The LAMB2 and LAMC1/C2/C3 mRNA expression levels were markedly correlated with worse PFS in grade 3 OC patients.

**TABLE 2:** The relationship between laminins and PFS in different tumor grades and stages of OC (Kaplan–Meier plotter). Bold font indicates significant difference.

Genes	Stage I + II (179 cases)		Stage III (1,079 cases)		Stage IV (189 cases)		Grade 1 + 2 (381 cases)		Grade 3 (1,024 cases)	
	HR (95%CI)	*p*-value	HR (95%CI)	*p*-value	HR (95%CI)	*p*-value	HR (95%CI)	*p*-value	HR (95%CI)	*p*-value
LAMA1	1.97	0.071	1.18	0.15	0.79	0.39	0.86	0.39	0.89	0.39
	(0.93–4.15)		(0.94–1.47)		(0.47–1.34)		(0.6–1.22)		(0.68–1.16)	
LAMA2	0.26	**0.00042**	1.19	0.059	1.16	0.46	0.59	**0.00025**	1.16	0.12
	(0.12–0.58)		(0.99–1.42)		(0.78–1.73)		(0.44–0.79)		(0.96–1.41)	
LAMA3	0.39	**0.00074**	0.87	0.067	1.81	**0.0018**	0.67	**0.0051**	1.1	0.26
	(0.22–0.69)		(0.74–1.01)		(1.24–2.64)		(0.5–0.89)		(0.93–1.31)	
LAMA4	0.4	**0.022**	**1.29**	**0.0014**	0.76	0.19	1.73	**0.00014**	1.22	**0.021**
	(0.18–0.9)		(1.1–1.5)		(0.5–1.15)		(1.3–2.3)		(1.03–1.44)	
LAMA5	2.65	**0.00096**	1.31	**0.00097**	1.84	**0.0014**	1.44	**0.014**	1.42	**8.7E−05**
	(1.45–4.83)		(1.12–1.54)		(1.26–2.68)		(1.07–1.93)		(1.19–1.7)	
LAMB1	0.5	**0.015**	1.18	**0.033**	1.3	0.18	1.16	0.31	1.1	0.26
	(0.28–0.88)		(1.01–1.38)		(0.89–1.9)		(0.87–1.54)		(0.93–1.3)	
LAMB2	0.73	0.29	1.22	**0.022**	1.31	0.15	1.16	0.35	1.25	**0.017**
	(0.41–1.31)		(1.03–1.44)		(0.91–1.9)		(0.85–1.58)		(1.04–1.49)	
LAMB3	0.55	0.08	1.09	0.32	1.53	0.053	0.74	0.079	1.1	0.33
	(0.28–1.08)		(0.92–1.3)		(0.99–2.35)		(0.53–1.04)		(0.91–1.32)	
LAMB4	0.72	0.25	1.13	0.19	0.76	0.18	0.84	0.22	1.08	0.42
	(0.41–1.27)		(0.94–1.34)		(0.51–1.13)		(0.63–1.12)		(0.9–1.28)	
LAMC1	0.73	0.29	1.22	**0.022**	1.31	0.15	1.16	0.35	1.25	**0.017**
	(0.41–1.31)		(1.03–1.44)		(0.91–1.9)		(0.85–1.58)		(1.04–1.49)	
LAMC2	0.42	**0.012**	1.09	0.36	1.22	0.33	0.64	**0.0031**	1.24	**0.034**
	(0.21–0.84)		(0.91–1.29)		(0.81–1.85)		(0.48–0.86)		(1.02–1.52)	
LAMC3	0.7	0.22	1.18	**0.033**	1.34	0.16	0.85	0.27	1.27	**0.0077**
	(0.39–1.24)		(1.01–1.38)		(0.89–2.02)		(0.63–1.14)		(1.07–1.52)	

Based on the OS and PFS results shown in Tables 1 and 2, upregulated expression levels of LAMA1/A4/A5 and LAMB1 may indicate poor prognosis in OC patients and LAMA5 might be considered the main negative prognostic factor for all stages and grades of OC. These results also suggest that LAMC2 expression may be used to predict a relatively longer prognosis for patients with OC.

### Diagnostic Evaluation of Laminins in Ovarian Cancer

We explored clinical data and information on laminin mRNA expression for OC patients in TCGA and extracted data from GTEx for the corresponding normal tissues. Data on 515 cases were available and the dataset was used to investigate the relationships of specific laminins via ROC (receiver operating characteristic) curve regression analysis. Our results were evaluated based on ROC curve AUC >0.70. We found that LAMA2 (AUC: 1.000; 95% CI: 0.999–1.000), LAMA4 (AUC: 0.881; 95% CI: 0.851–0.912), LAMA5 (AUC: 0.894; 95% CI: 0.859–0.928), LAMB1 (AUC: 0.889; 95% CI: 0.854–0.924), LAMB2 (AUC: 0.994; 95% CI: 0.990–0.999), LAMB3 (AUC: 0.977; 95% CI: 0.955–0.999), and LAMC2 (AUC: 0.992; 95% CI: 0.985–0.999) were qualified (Supplementary Figure S3).

Overall, the ROC analysis of the laminins in the OC sets indicated that LAMA2/A4/A5, LAMB1/B2/B3, and LAMC2 could be considered to accurately differentiate between tumor and non-tumor tissues.

### Association Between Laminin Expression and Immune Cell Infiltration in Ovarian Cancer

**FIGURE 5:**
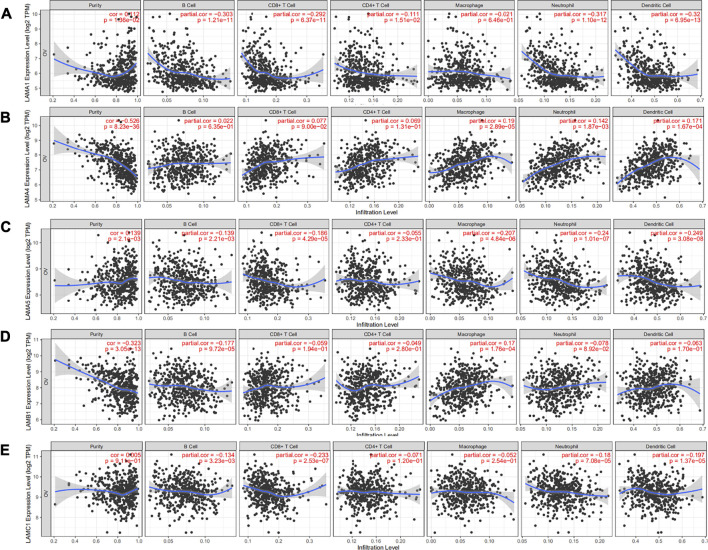
Correlation analysis of laminin genes and immunocyte infiltration levels in OC tissues. **(A)** Correlation between LAMA1 mRNA and immunocyte infiltration levels **(B)** Correlation between LAMA4 mRNA and immunocyte infiltration levels. **(C)** Correlation between LAMA5 mRNA and immunocyte infiltration levels **(D)** Correlation between LAMB1 mRNA and immunocyte infiltration levels **(E)** Correlation between LAMC1 mRNA and immunocyte infiltration levels.

Epithelial ovarian cancer (EOC) is susceptible to immune recognition[26]. CD8+TIL expression is associated with better prognosis in advanced EOC patients[27]. Based on the existing literature, we used the online TIMER database to explore associations between laminins and immune infiltration levels in OC patients. There were negative correlations between OC tumor purity and LAMA4 and LAMB1. The correlation ratio was the most considerable for LAMA4 (r = −0.526; *p* = 8.23E-36). LAMA4 was positively weakly correlated with macrophage (r = 0.19; *p* = 2.89E-05), neutrophil (r = 0.142; *p* = 1.87E-03) and dendritic cell (r = 0.171; *p* = 1.64E-04), while LAMB1 was positively weakly correlated with only macrophage (r = 0.17; *p* = 1.76E-04). Details of the relationships between immune cell types and laminin members are illustrated in Figure 5 and Supplementary Figure S4.

LAMA1 was negatively correlated with CD8+ T cell (r = −0.692; *p* = 6.37E-11), B cell (r = −0.303; *p* = 1.21E-11), neutrophil (r = −0.317; *p* = 1.10E-12) and dendritic cell (r = −0.32; *p* = 6.95E-13). LAMA5 was significantly and negatively correlated with macrophage (r = −0.207; *p* = 4.84E-06), neutrophil (r = −0.24; *p* = 1.01E-7) and dendritic cell (r = −0.249; *p* = 3.08E-08). LAMC1 was significantly and negatively correlated with CD8+ T cell (r = −0.233; *p* = 2.53E-07). However, LAMA2/A3, LAMB2/B3/B4, and LAMC2/C3 were not associated with any specific immune cell types (Supplementary Figure S4).

### Relationship Between Laminin Genes and Ovarian Cancer Metastasis

The development of platinum resistance in tumors leads to inferior outcomes and remains an obstacle in efficacious anticancer therapy. To investigate whether laminin genes could be implicated in the development of endogenous chemotherapy resistance, we retrieved data from GEO regarding the responses of OC patients to chemotherapy. GSE131978 defined patients with tumor progression times >12 months as being relatively platinum-sensitive and those with tumor progression times < six months as being platinum-resistant. LAMA1 and LAMB3 expression levels were upregulated in platinum-resistant OC patients (Supplementary Table S2). We also explored the GSE58470 dataset[28]. It includes the parental cisplatin-sensitive IGROV-1 and the platinum-resistant variant IGROV-1/Pt1 OC cell lines. LAMC2 expression was significantly upregulated in the platinum-resistant mutants (Supplementary Table S2). Hence, LAMA1, LAMB3, and LAMC2 may play important roles in the development of platinum resistance.

### Kyoto Encyclopedia of Genes and Genomes Pathway, Gene Ontology Term, and Interaction Network Analyses of Laminin Family Genes

**FIGURE 6:**
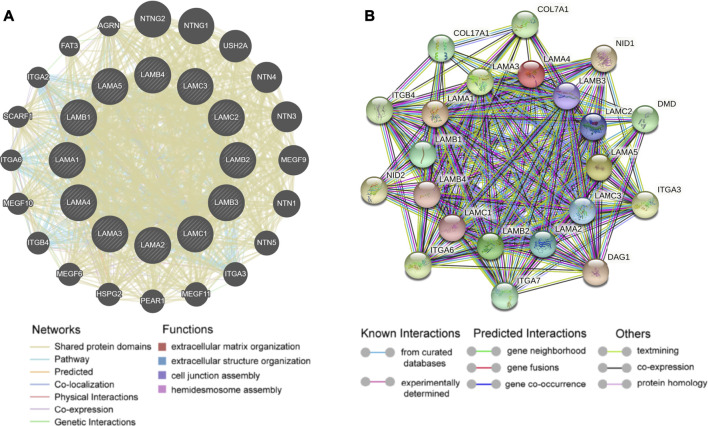
Gene-gene interaction and protein-protein interaction network of laminin genes **(A)** The laminin gene family network; illustration generated using GeneMANIA. Colored patches on circle indicate gene function **(B)** Network diagram of interactions between proteins encoded by laminin family genes; illustration generated using STRING.

In the gene interaction network generated based on the use of GeneMANIA[29], we observed that laminin family genes might be associated with ITG and netrin family genes (Figure 6A). We used STRING[25] to analyze the protein-protein interaction network (local clustering coefficient = 0.875). Laminin proteins were co-expressed with ITG, nidogen, dystrophin, dystroglycan, collagen types XVII and VII, and alpha 1 chain proteins (Figure 6B).

The GO and KEGG databases revealed laminin functions and the genes that were significantly associated with their alterations. Biological processes such as GO:0030198 (extracellular matrix organization), GO:0001568 (blood vessel development), and others were remarkably influenced by laminin mutations in OC (Supplementary Figure S5A). Molecular functions such as GO:0004060 (arylamine *N*-acetyltransferase activity), GO:0001228 (DNA-binding transcription activator activity, RNA polymerase II-specific), GO:0005201 (extracellular matrix structural constituent), GO:0005509 (calcium ion binding), GO:0005518 (collagen binding), GO:0050839 (cell adhesion molecule binding), and others were significantly associated with laminin alterations (Supplementary Figure S5B). Laminin mutations also significantly affected cellular components including GO:0031012 (extracellular matrix), GO:0070161 (anchoring junction), and GO:0005938 (cell cortex) (Supplementary Figure S5C). In the KEGG analysis, ten pathways including ko04512 (ECM-receptor interaction), ko04010 (MAPK signaling pathway), ko05412 (arrhythmogenic right ventricular cardiomyopathy (ARVC)), ko04210 (apoptosis), and others were affected by laminin mutations in OC (Supplementary Figure S5D).

## Discussion

Our study demonstrated that certain OC patients presented with laminin mutations. The mutation frequencies were as follows: LAMA5, 30%; LAMC1, 16%; LAMC2, 12%; LAMA3, 11%; and LAMB1, 11%. Laminin mutation was associated with poor prognosis in ovarian cancer. The LAMA5, LAMB3, and LAMC2 mRNA expression levels were higher in OC than those in normal ovary tissues and LAMA2/A4 and LAMB1 were downregulated in OC tissues, while the LAMA3, LAMB1/B2/B3, and LAMC1/C2 protein expression levels were higher in OC than those in normal ovary tissues. A survival analysis demonstrated that upregulated levels of LAMA4, LAMB1, and LAMC1 mRNAs were positively associated with poor OS and PFS in OC patients. In OC, LAMA5 overexpression was related to poor prognosis in advanced OS, high-grade OS, and PFS at all stages and grades. However, elevated LAMA2 and LAMC2 mRNA expression levels were associated with better PFS in early-stage OC patients. LAMB3 and LAMC2 expression was significantly related to platinum resistance development in OC patients. The ROC analysis of the laminins in OC datasets showed that the LAMA2/A4/A5, LAMB1/B2/B3, and LAMC2 mRNA levels could be considered to effectively differentiate between malignant tumor and non-tumor tissues. LAMA4/A5, LAMB1, and LAMC1 may perform multiple functions in immunocyte infiltration in OC. LAMA4 and LAMB1 may be implicated in tumor purity.

In the present study, we found that LAMA5 might be crucial to OC occurrence and progression. Compared with normal ovary tissues, LAMA5 was the most frequently altered gene (30%) and was overexpressed at mRNA level in OC patients. Moreover, LAMA5 was associated with poor PFS in all stages and grades and poor OS in stage Ⅳ and grade 3 OC. Bartolini et al. reported that LAMA5 was a molecular target in colorectal cancer cells with the KRAS mutation. Furthermore,the tumor microenvironment (TME) was overexpressed[9]. Gordon-Weeks et al. have reported that LAMA5 is necessary for liver metastasis as it promotes branch angiogenesis and modulates Notch signaling[12]. Maltseva D et al. demonstrated that LAMA5 knockdown led to Wnt- and mTORC1-dependent partial dedifferentiation of colorectal carcinoma which, in turn, was related to the activation of endoplasmic reticulum stress (ERS) signaling pathways promoting 5-fluorouracil (5-FU) sensitivity. Thus, LAMA5 is deemed a potential therapeutic antitumor target[30]. A recent study has shown that LAMA5 binds integrin-6 and promotes epithelial-to-mesenchymal transition (EMT). This research also identified LAMA5 as a putative antitumor target[30]. Nevertheless, the mechanism by which LAMA5 promotes OC has not been elucidated and merits further investigation *in vivo* and *in vitro*.

We also identified other laminin family members involved in OC progression. The mutation frequency of LAMC1 was 16% and it ranked second only to LAMA5. The LAMC1 mRNA and protein expressions levels were increased in OC compared to those in normal ovary tissue and indicated a worse prognosis for OC patients. RNA sequencing was performed to compare the transcriptomes between high-grade serous carcinomas (HGSC) and normal fallopian tube epithelia (FTE). Findings revealed that LAMC1 expression upregulation was associated with HGSC[31]. Kunitomi et al. stated that LAMC1 overexpression could be considered an effective biomarker in endometrial cancer patients requiring aggressive adjuvant therapy[32]. According to Zhang et al., LAMC1 overexpression indicates poor prognosis in hepatocellular carcinoma (HCC)[33]. LAMC1 overexpression also indicated poor prognosis for cutaneous squamous cell carcinoma[34] and prostate cancer[35]. LAMC1 may participate in HCC progression by regulating PKM2 expression via the PTEN/AKT pathway[6]. Nevertheless, the pathways affected by LAMC1 in ovarian cancer remain to be determined.

Our study speculated that the role of LAMC2 in the progression of OC remains to be validated. The mutation frequency of LAMC2 is 12% in OC and LAMC2 expression is upregulated in OC tissues. LAMC2 expression was upregulated in SKOV-3 (ovarian serous carcinoma cell line) compared with MCV152 (benign ovarian epithelial tumor line)[36]. LAMC2 expression upregulation was also confirmed in OC tissues and cells. It promoted OC cell proliferation and inhibited OC cell its apoptosis via the miR-125a-5p/LAMC2/p38 pathway[37]. Moreover, LAMC2 expression upregulation lowered the sensitivity of OC cells to the chemotherapeutic agents docetaxel and taxane via the PI3K/Akt axis[38]. LAMC2 expression upregulation was usually associated with worse prognosis for patients with head and neck squamous cell carcinoma (HNSC) [39], colorectal cancer[40], lung adenocarcinoma[41] and bladder cancer[42]. However, our bioinformatics analysis in the Kaplan-Meier plotter showed that OC patients with upregulated LAMC2 expression levels had better PFS and OS than those with low LAMC2 expression levels. The underlying explanation for this discrepancy must be clarified through large-scale clinical studies.

Our bioinformatics analysis suggested that several other laminin family members were associated with OC progression. LAMA1 overexpression was significantly associated with poor prognosis and platinum resistance development in OS. The LAMA2 and LAMA4 mRNA expression levels were relatively lower in OC tissues. However, a survival analysis showed that PFS worsened with increasing LAMA4 expression levels in OC patients. ROC analysis revealed that LAMA2/A4/A5, LAMB1/B2/B3, and LAMC2 could be considered to effectively distinguish OC patients from healthy subjects. The foregoing findings may provide new insights into targets for antitumor therapy and early diagnosis in OC.

Immunotherapy is a novel frontier in the treatment of recurrent EOC[27]. Approximately half of all advanced EOC patients presented with spontaneous antitumor response evidenced by the generation of tumor-specific lymphocytes and antibodies[26]. Our analysis speculated that LAMA1/A4/A5, LAMB1, and LAMC1 were associated with the immune infiltration levels of OC. Li et al. found that LAMA5 in murine lymph node stromal cells (LNSCs) modulated the LN immune response by changing LN structure and T cell behavior[43]. LAMA4 and LAMA5 demonstrated complex formation necessary for regulating human and mouse CD4 T cells[44]. In mice, LAMA4 expression downregulation weakened the immune cells penetrating vessel walls[45]. In head and neck squamous cell carcinoma, LAMC2 expression was strongly associated with B cell, CD8+ T cell, CD4^+^ T cell, and macrophage infiltration levels[39]. The preceding discoveries may provide clues for immunotherapy in OC patients.

The present study had certain limitations. First, it depended on data retrieved from continuously monitored and expanded online databases. Hence, these changes could have influenced the results of this study. Studies with large sample sizes are warranted to validate our findings and to assess the feasibility of clinical laminin application as an OC treatment. Second, we did not investigate the indirect or direct mechanisms of different laminin proteins in OC. Future studies should be conducted to explore the specific modes of action of the different laminin members in OC. We plan to further study the role of laminins in OC *in vitro* in OC cell lines in terms of basic phenotypes such as cell cycle, apoptosis, and senescence and *in vivo* by constructing OC animal models.

The results of this research suggested that LAMA5 and LAMC1 might be considered important OC oncogenes and prognostic factors in OC. The present study also indicated that LAMA1/A4/A5, LAMB4, and LAMC1 might play important roles in the OC immune response and could be used to improve immunotherapy efficacy in OC. It is hoped that our findings will help improve treatment efficacy and prognostic accuracy in OC.

## Data Availability

The original contributions presented in the study are included in the article/Supplementary Material, further inquiries can be directed to the corresponding author.
