# Corrigendum: Comprehensive Analysis of the Expression and Prognosis for Laminin Genes in Ovarian Cancer

**DOI:** 10.3389/pore.2022.1610258

**Published:** 2022-02-18

**Authors:** Bowen Diao, Ping Yang

**Affiliations:** Department of Gynecology, First Affifiliated Hospital, School of Medicine, Shihezi University, Shihezi, China

**Keywords:** ovarian cancer, metastasis, laminins, cancer prognosis, bioinformatical analysis

There is an error in the **Funding** statement. The correct number for National Natural Science Foundation of China is “82072893”.

In the original article, there was an error. In the results section, the database name in the first paragraph was incorrect. A correction has been made to **Results, Genetic Mutations in Laminins and Their Associations With Overall Survival and Disease-free Survival in Patients With Ovarian Cancer**, paragraph one:

“We evaluated genetic alterations in laminins and their relationships with the OS and DFS of OC patients registered in the cBioPortal database”.

The authors apologize for this error and state that this does not change the scientific conclusions of the article in any way. The original article has been updated.

