# Supplementary material for: Comprehensive Analysis of the Expression and Prognosis for Laminin Genes in Ovarian Cancer
Source: Pathol Oncol Res. 2021 Aug 25;27:1609855. doi: 10.3389/pore.2021.1609855 (PMC8423899; doi:10.3389/pore.2021.1609855)
Supplement: Supplementary file 1 [file DataSheet1.zip › Supplementary material/Supplementary Table 1.docx]

Supplementary Table 1 Significant changes of laminins expression in transcription level between OC and normal tissues (ONCOMINE).

|  | Type of Ovarian Cancer versus Normal Tissue | Fold Change | p Value | t Test | Source and/or Reference |
| --- | --- | --- | --- | --- | --- |
| LAMA2 | Ovarian Serous Surface Papillary Carcinoma vs. Normal | -32.074 | 4.54E-12 | -11.078 | (Welsh et al., 2001) |
|  | Ovarian Serous Adenocarcinoma vs. Normal | -14.229 | 2.70E-19 | -14.086 | (Yoshihara et al., 2009) |
|  | Ovarian Serous Adenocarcinoma vs. Normal | -6.471 | 1.83E-4 | -5.945 | (Adib et al., 2004) |
|  | Ovarian Mucinous Adenocarcinoma vs. Normal | -1.834 | 1.38E-7 | -10.010 | (Hendrix et al., 2006) |
|  | Ovarian Clear Cell Adenocarcinoma vs. Normal | -1.764 | 4.56E-6 | -9.562 | (Hendrix et al., 2006) |
|  | Ovarian Endometrioid Adenocarcinoma vs. Normal | -1.819 | 1.04E-7 | -11.938 | (Hendrix et al., 2006) |
|  | Ovarian Serous Adenocarcinoma vs. Normal | -2.053 | 3.77E-7 | -15.962 | (Hendrix et al., 2006) |
|  | Ovarian Serous Cystadenocarcinoma vs. Normal | -4.369 | 3.81E-6 | -11.243 | TCGA Ovarian Statistics |
|  | Ovarian Carcinoma vs. Normal | -1.617 | 0.003 | -3.459 | (Bonome et al., 2008) |
| LAMA3 | Ovarian Mucinous Adenocarcinoma vs. Normal | 1.900 | 0.004 | 3.203 | (Lu et al., 2004) |
|  | Ovarian Serous Adenocarcinoma vs. Normal | 1.530 | 0.007 | 2.739 | (Lu et al., 2004) |
|  | Ovarian Serous Surface Papillary Carcinoma vs. Normal | 21.254 | 0.003 | 3.752 | (Welsh et al., 2001) |
|  | Ovarian Serous Cystadenocarcinoma vs. Normal | -2.385 | 0.002 | -4.208 | TCGA Ovarian Statistics |
| LAMA4 | Ovarian Serous Adenocarcinoma vs. Normal | -7.234 | 1.01E-16 | -13.273 | (Yoshihara et al., 2009) |
|  | Ovarian Carcinoma vs. Normal | -6.807 | 2.42E-7 | -11.051 | (Bonome et al., 2008) |
|  | Ovarian Serous Cystadenocarcinoma vs. Normal | -1.845 | 8.34E-4 | -4.699 | TCGA Ovarian Statistics |
| LAMA5 | Ovarian Clear Cell Adenocarcinoma vs. Normal | 2.330 | 9.58E-9 | 16.571 | (Hendrix et al., 2006) |
|  | Ovarian Endometrioid Adenocarcinoma vs. Normal | 1.782 | 3.58E-11 | 15.199 | (Hendrix et al., 2006) |
|  | Ovarian Serous Adenocarcinoma vs. Normal | 1.937 | 1.29E-8 | 21.414 | (Hendrix et al., 2006) |
|  | Ovarian Clear Cell Adenocarcinoma vs. Normal | 3.162 | 2.24E-4 | 5.330 | (Lu et al., 2004) |
|  | Ovarian Serous Adenocarcinoma vs. Normal | 2.280 | 7.03E-5 | 4.973 | (Lu et al., 2004) |
|  | Ovarian Endometrioid Adenocarcinoma vs. Normal | 1.707 | 0.003 | 3.533 | (Lu et al., 2004) |
|  | Ovarian Carcinoma vs. Normal | 1.542 | 4.71E-6 | 5.681 | (Bonome et al., 2008) |
|  | Ovarian Serous Cystadenocarcinoma vs. Normal | 1.943 | 1.33E-5 | 8.505 | TCGA Ovarian Statistics |
|  | Ovarian Serous Adenocarcinoma vs. Normal | 2.158 | 0.015 | 2.680 | (Adib et al., 2004) |
|  | Ovarian Serous Adenocarcinoma vs. Normal | 1.712 | 0.005 | 2.798 | (Yoshihara et al., 2009) |
| LAMB1 | Ovarian Clear Cell Adenocarcinoma vs. Normal | 1.794 | 6.06E-5 | 10.166 | (Hendrix et al., 2006) |
|  | Ovarian Serous Cystadenocarcinoma vs. Normal | 2.536 | 5.54E-5 | 7.004 | TCGA Ovarian Statistics |
|  | Ovarian Serous Adenocarcinoma vs. Normal | -4.105 | 9.85E-7 | -5.968 | (Yoshihara et al., 2009) |
|  | Ovarian Carcinoma vs. Normal | -4.882 | 1.83E-4 | -5.360 | (Bonome et al., 2008) |
| LAMB2 | Ovarian Serous Adenocarcinoma vs. Normal | -1.875 | 1.37E-4 | -6.508 | (Adib et al., 2004) |
|  | Ovarian Serous Surface Papillary Carcinoma vs. Normal | -2.465 | 2.09E-6 | -6.143 | (Welsh et al., 2001) |
|  | Ovarian Serous Adenocarcinoma vs. Normal | -2.784 | 1.22E-8 | -7.905 | (Yoshihara et al., 2009) |
| LAMB3 | Ovarian Serous Surface Papillary Carcinoma vs. Normal | 98.621 | 5.44E-17 | 18.192 | (Welsh et al., 2001) |
|  | Ovarian Mucinous Adenocarcinoma vs. Normal | 1.906 | 8.27E-10 | 12.896 | (Hendrix et al., 2006) |
|  | Ovarian Serous Adenocarcinoma vs. Normal | 1.519 | 4.37E-10 | 9.024 | (Hendrix et al., 2006) |
|  | Ovarian Clear Cell Adenocarcinoma vs. Normal | 1.645 | 1.73E-5 | 7.682 | (Hendrix et al., 2006) |
|  | Ovarian Serous Adenocarcinoma vs. Normal | 11.103 | 3.92E-8 | 8.736 | (Yoshihara et al., 2009) |
|  | Ovarian Mucinous Adenocarcinoma vs. Normal | 2.005 | 0.002 | 3.735 | (Lu et al., 2004) |
|  | Ovarian Clear Cell Adenocarcinoma vs. Normal | 1.642 | 0.016 | 2.545 | (Lu et al., 2004) |
|  | Ovarian Serous Adenocarcinoma vs. Normal | 1.851 | 0.006 | 3.297 | (Adib et al., 2004) |
|  | Ovarian Mucinous Adenocarcinoma vs. Normal | -4.369 | 9.26E-4 | -1.573 | (Lu et al., 2004) |
| LAMC1 | Ovarian Clear Cell Adenocarcinoma vs. Normal | 1.792 | 2.45E-6 | 8.845 | (Hendrix et al., 2006) |
|  | Ovarian Clear Cell Adenocarcinoma vs. Normal | 3.341 | 6.29E-4 | 4.439 | (Lu et al., 2004) |
|  | Ovarian Carcinoma vs. Normal | 2.119 | 7.55E-11 | 13.400 | (Bonome et al., 2008) |
|  | Ovarian Serous Cystadenocarcinoma vs. Normal | 2.324 | 1.26E-6 | 11.058 | TCGA Ovarian Statistics |
|  | Ovarian Serous Adenocarcinoma vs. Normal | -2.626 | 4.07E-10 | -8.440 | (Yoshihara et al., 2009) |
| LAMC2 | Ovarian Serous Adenocarcinoma vs. Normal | 1.627 | 1.86E-4 | 5.911 | (Adib et al., 2004) |
|  | Ovarian Serous Adenocarcinoma vs. Normal | 1.556 | 8.09E-10 | 10.052 | (Hendrix et al., 2006) |
|  | Ovarian Mucinous Adenocarcinoma vs. Normal | 1.707 | 7.84E-7 | 7.730 | (Hendrix et al., 2006) |
|  | Ovarian Serous Adenocarcinoma vs. Normal | 7.857 | 1.17E-6 | 7.186 | (Yoshihara et al., 2009) |
| TCGA, The Cancer Genome Atlas | |  |  |  |  |

Adib, T., Henderson, S., Perrett, C., Hewitt, D., Bourmpoulia, D., Ledermann, J., & Boshoff, C. J. B. j. o. c. (2004). Predicting biomarkers for ovarian cancer using gene-expression microarrays. *90*(3), 686-692. doi:10.1038/sj.bjc.6601603

Bonome, T., Levine, D., Shih, J., Randonovich, M., Pise-Masison, C., Bogomolniy, F., . . . Birrer, M. J. C. r. (2008). A gene signature predicting for survival in suboptimally debulked patients with ovarian cancer. *68*(13), 5478-5486. doi:10.1158/0008-5472.Can-07-6595

Hendrix, N., Wu, R., Kuick, R., Schwartz, D., Fearon, E., & Cho, K. J. C. r. (2006). Fibroblast growth factor 9 has oncogenic activity and is a downstream target of Wnt signaling in ovarian endometrioid adenocarcinomas. *66*(3), 1354-1362. doi:10.1158/0008-5472.Can-05-3694

Lu, K., Patterson, A., Wang, L., Marquez, R., Atkinson, E., Baggerly, K., . . . Bast, R. J. C. c. r. a. o. j. o. t. A. A. f. C. R. (2004). Selection of potential markers for epithelial ovarian cancer with gene expression arrays and recursive descent partition analysis. *10*(10), 3291-3300. doi:10.1158/1078-0432.Ccr-03-0409

Welsh, J., Zarrinkar, P., Sapinoso, L., Kern, S., Behling, C., Monk, B., . . . Hampton, G. J. P. o. t. N. A. o. S. o. t. U. S. o. A. (2001). Analysis of gene expression profiles in normal and neoplastic ovarian tissue samples identifies candidate molecular markers of epithelial ovarian cancer. *98*(3), 1176-1181. doi:10.1073/pnas.98.3.1176

Yoshihara, K., Tajima, A., Komata, D., Yamamoto, T., Kodama, S., Fujiwara, H., . . . Tanaka, K. J. C. s. (2009). Gene expression profiling of advanced-stage serous ovarian cancers distinguishes novel subclasses and implicates ZEB2 in tumor progression and prognosis. *100*(8), 1421-1428. doi:10.1111/j.1349-7006.2009.01204.x
