# Supplementary material for: Comprehensive Analysis of the Expression and Prognosis for Laminin Genes in Ovarian Cancer
Source: Pathol Oncol Res. 2021 Aug 25;27:1609855. doi: 10.3389/pore.2021.1609855 (PMC8423899; doi:10.3389/pore.2021.1609855)
Supplement: Supplementary file 1 [file DataSheet1.zip › Supplementary material/Supplementary table2.docx]

Supplementary Table 2 Significant changes of laminins expression in OC tissues’ and OC cell lines’ transcription level from GEO database.

|  | Gene | p Value | Fold Change | Overall design |
| --- | --- | --- | --- | --- |
| GSE131978 | LAMA1 | 8.28E-03 | -1.52 | Resistant vs. Sensitive OC tissues |
|  | LAMB3 | 4.50E-02 | -1.74 |  |
| GSE58470 | LAMC2 | 1.18E-02 | -1.18 | The parental cisplatin-sensitive IGROV-1 cell line vs. The platinum-resistant variants IGROV-1/Pt1 |
