# Supplementary figures and images for: Comprehensive Analysis of the Expression and Prognosis for Laminin Genes in Ovarian Cancer
Source: Pathol Oncol Res. 2021 Aug 25;27:1609855. doi: 10.3389/pore.2021.1609855 (PMC8423899; doi:10.3389/pore.2021.1609855)

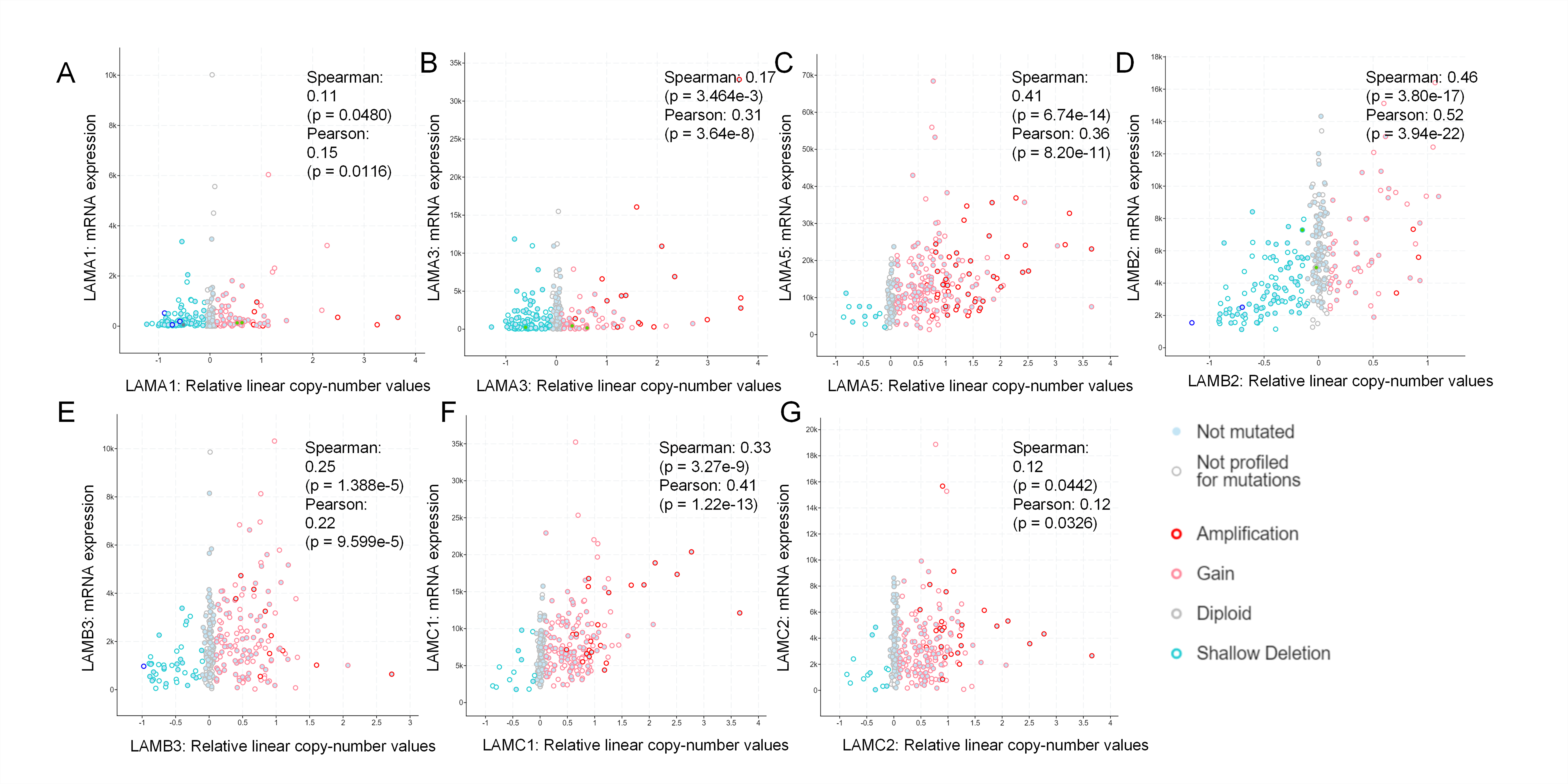

Supplement: Supplementary file 1 [file DataSheet1.zip › Supplementary material/Supplementary Figure1.tif]

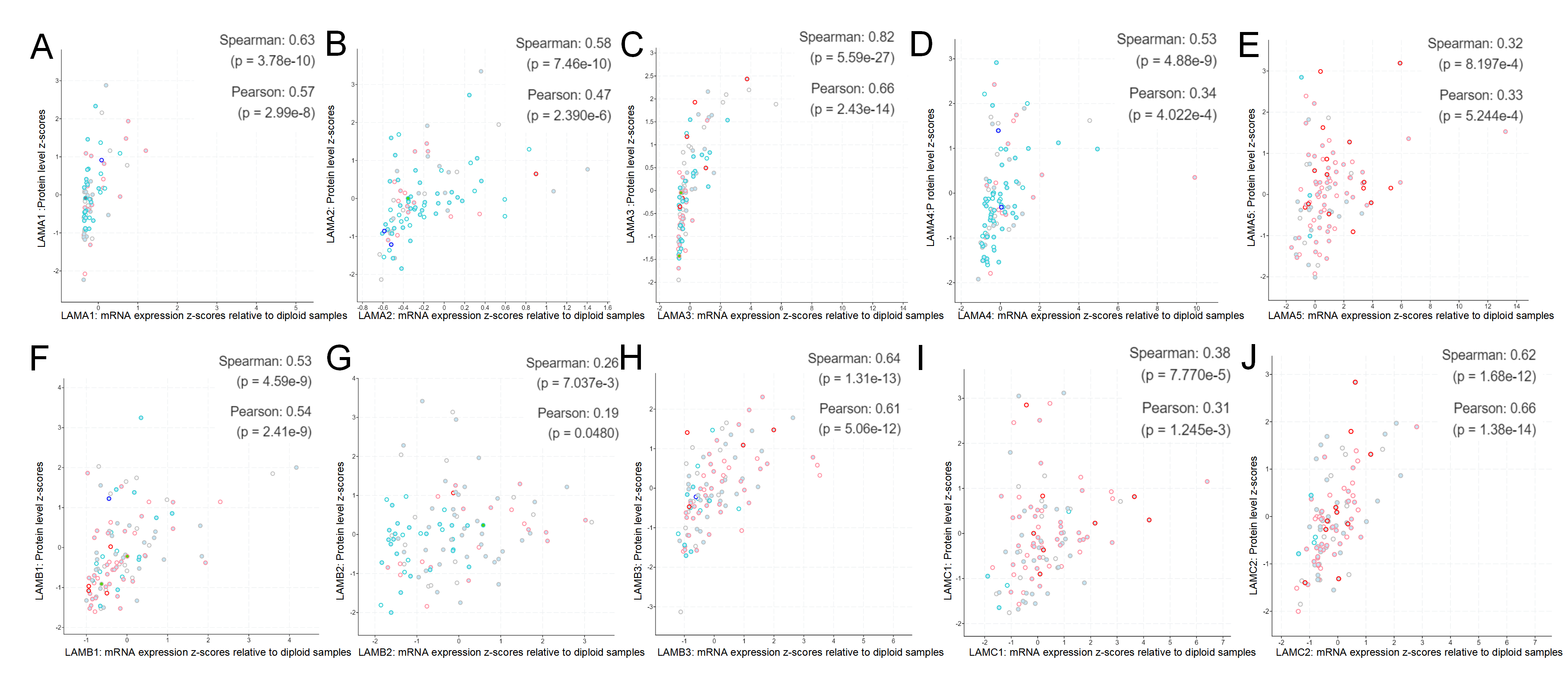

Supplement: Supplementary file 1 [file DataSheet1.zip › Supplementary material/Supplementary Figure2.tif]

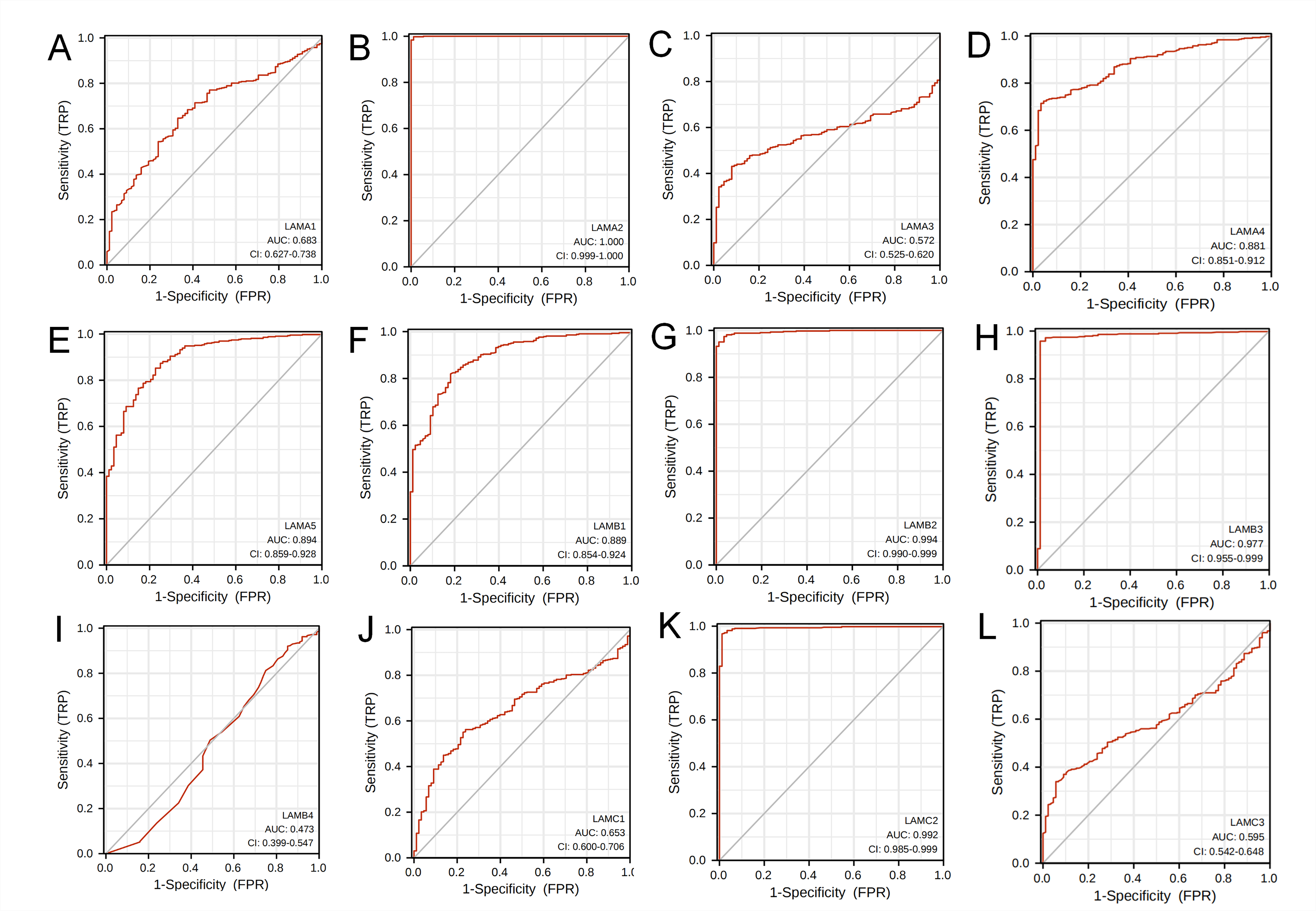

Supplement: Supplementary file 1 [file DataSheet1.zip › Supplementary material/Supplementary Figure3.tif]

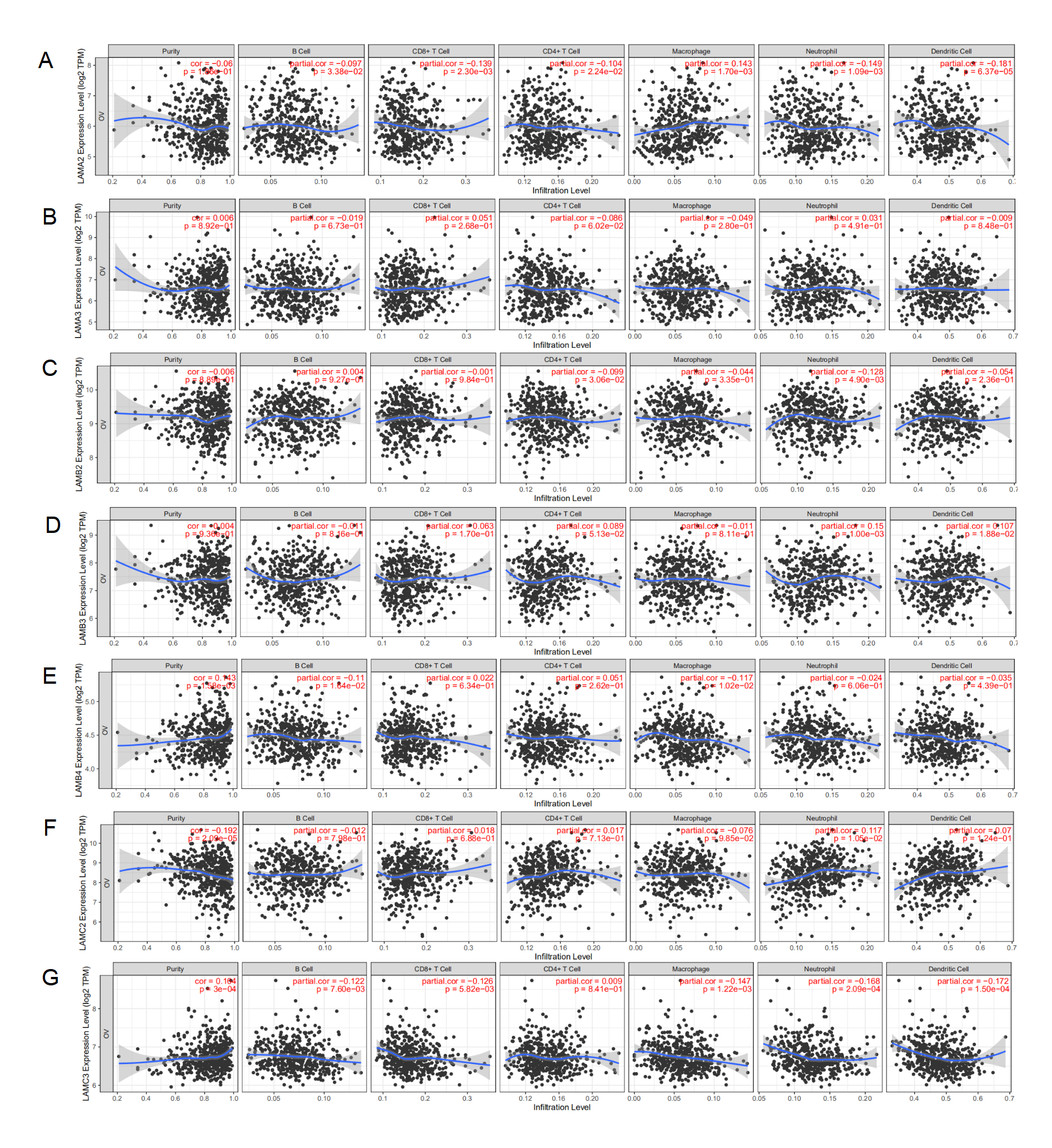

Supplement: Supplementary file 1 [file DataSheet1.zip › Supplementary material/Supplementary Figure4.tif]

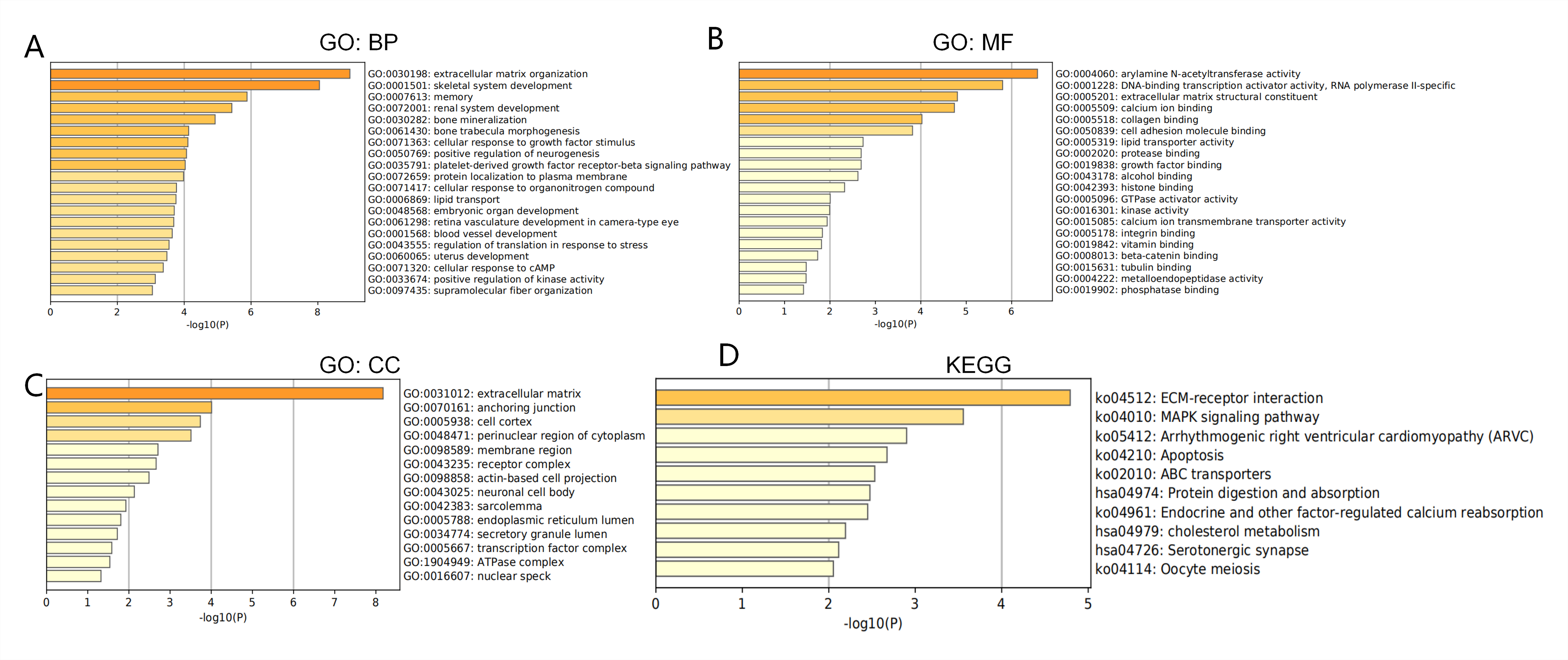

Supplement: Supplementary file 1 [file DataSheet1.zip › Supplementary material/Supplementary Figure5.tif]
